# Distribution and genetic diversity of Enterovirus G (EV-G) on pig farms in Thailand

**DOI:** 10.1186/s12917-021-02988-6

**Published:** 2021-08-16

**Authors:** Taveesak Janetanakit, Supassama Chaiyawong, Kamonpan Charoenkul, Ratanaporn Tangwangvivat, Ekkapat Chamsai, Kitikhun Udom, Waleemas Jairak, Alongkorn Amonsin

**Affiliations:** 1 Center of Excellence for Emerging and Re-emerging Infectious Diseases in Animals and One Health Research Cluster, Faculty of Veterinary Science, Chulalongkorn University, Bangkok, Thailand; 2 Department of Veterinary Public Health, Faculty of Veterinary Science, Chulalongkorn University, Bangkok, 10330 Thailand

**Keywords:** Distribution, Diversity, Enterovirus G, Pigs, Thailand

## Abstract

**Background:**

Enterovirus G (EV-G) causes subclinical infections and is occasionally associated with diarrhea in pigs. In this study, we conducted a cross-sectional survey of EV-G in pigs from 73 pig farms in 20 provinces of Thailand from December 2014 to January 2018.

**Results:**

Our results showed a high occurrence of EV-Gs which 71.6 % of fecal and intestinal samples (556/777) and 71.2 % of pig farms (52/73) were positive for EV-G by RT-PCR specific to the 5’UTR. EV-Gs could be detected in all age pig groups, and the percentage positivity was highest in the fattening group (89.7 %), followed by the nursery group (89.4 %). To characterize the viruses, 34 EV-G representatives were characterized by VP1 gene sequencing. Pairwise sequence comparison and phylogenetic analysis showed that Thai-EV-Gs belonged to the EV-G1, EV-G3, EV-G4, EV-G8, EV-G9 and EV-G10 genotypes, among which the EV-G3 was the predominant genotype in Thailand. Co-infection with different EV-G genotypes or with EV-Gs and porcine epidemic diarrhea virus (PEDV) or porcine deltacoronavirus (PDCoV) on the same pig farms was observed.

**Conclusions:**

Our results confirmed that EV-G infection is endemic in Thailand, with a high genetic diversity of different genotypes. This study constitutes the first report of the genetic characterization of EV-GS in pigs in Thailand.

**Supplementary Information:**

The online version contains supplementary material available at 10.1186/s12917-021-02988-6.

## Background

Porcine enterovirus (PEV) infection is an important viral disease of pigs, causing swine production losses due to subclinical infections and gastroenteritis disorders. PEV belongs to the family *Picornaviridae*, genus *Enterovirus*. PEVs were originally classified into 13 types (PEV-1 to PEV-13). Then, PEV-1 to 7 and PEV-11 to 13 were reclassified to the genus *Teschovirus*, and PEV-8 was reclassified to the genus *Sapelovirus* (PSV). PEV-9 to 10 were reclassified as Enterovirus species G (EV-G) [1, 2]. To date, viruses within species EV-G (EV-Gs) have been classified into more than 20 genotypes [3–8]. The prototypes of EV-Gs are EV-G1 (previously named PEV9) and EV-G2 (previously named PEV10).

EV-Gs is a small nonenveloped positive-sense single-stranded RNA virus. The virus has only one open reading frame encoding viral polyproteins. Its genome organization includes a 5’UTR, P1, P2, P3 and a 3’UTR. After translation, the P1 protein can be cleaved into 4 structural proteins, VP1, VP2, VP3, and VP4. P2 and P3 can be cleaved into 7 nonstructural proteins, including 2Apro, 2B, 2C, 3A, 3B (VPg), 3Cpro, and 3Dpol (RdRp). It has been reported that pigs could be infected with enterovirus G (EV-G), porcine Teschovirus (PTV), porcine Sapelovirus (PSV) and also human enteroviruses, especially EV-A71. EV-A71 is a member of enterovirus species A and causes hand foot and mouth disease in children [9, 10]. In an experimental setting, pigs could be infected with EV-A71 and develop clinical signs [11].

EV-Gs can be found in both healthy pigs and in pigs with diarrhea. Mostly, infected pigs are subclinically infected [12, 13]. EV-Gs have been reported in pigs in Brazil, China, Japan, Korea, Hungary, and the USA and in wild boars in Hungary. There is limited information on systemic infection of EV-Gs in pigs that develop clinical conditions, including dermatitis, flaccid paralysis and diarrhea [14–16]. Recently, EV-Gs containing papain-like cysteine protease (EV-G-PL^pro^) have been reported to be associated with enteric diseases in pigs [17]. The co-circulation of multiple EV-G genotypes and recombination among EV-G genotypes has also been documented [18]. Due to the limited information on the occurrences and status of EV-Gs, we carried out a survey and genetic characterization of EV-Gs on pig farms from 7 livestock regions of Thailand during December 2014 – January 2018.

## Results

**Fig. 1:**
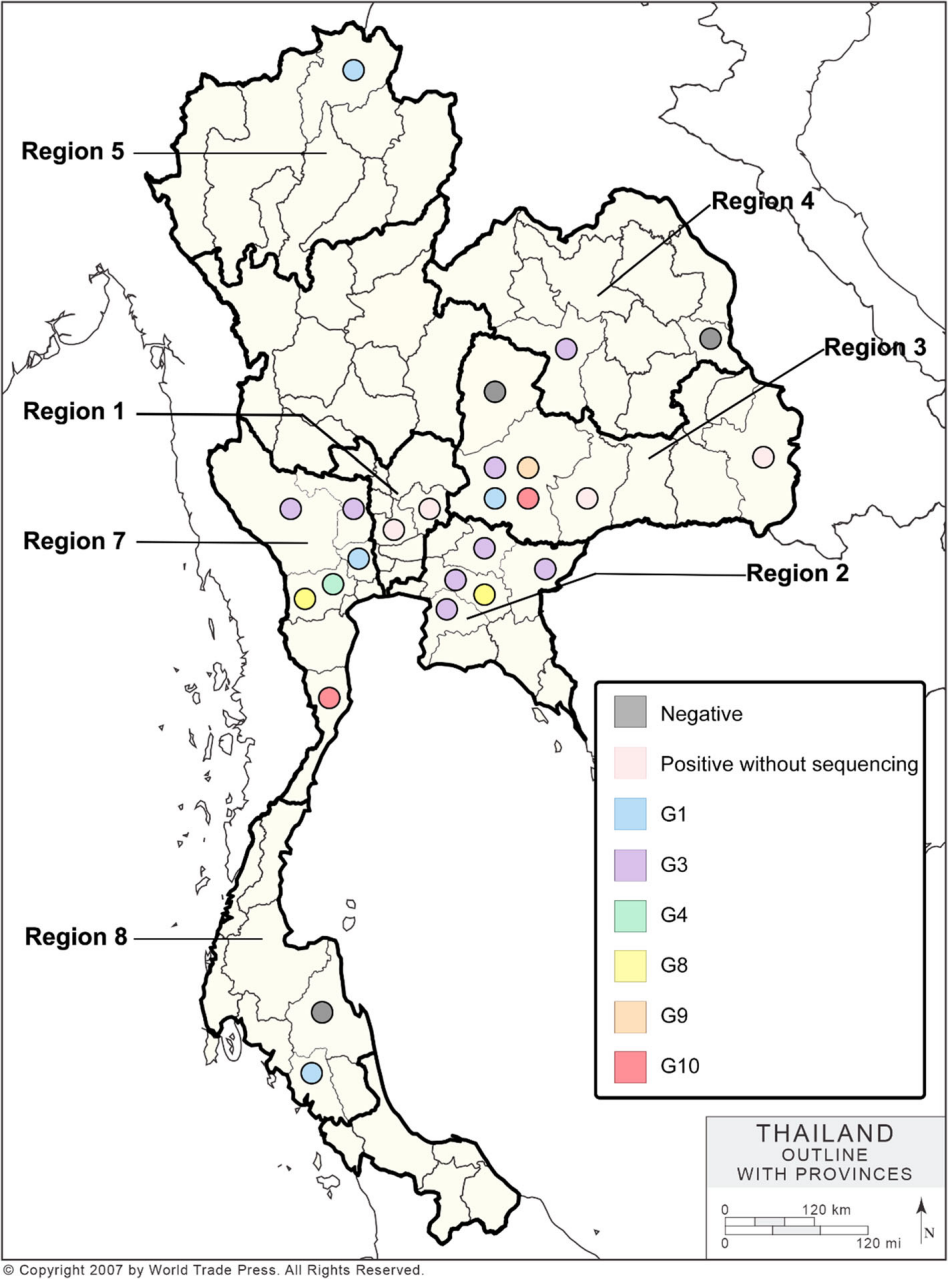
Distribution of the Thai-EV-Gs by genotype. Outline areas represent livestock region of Thailand included in this study. Each color circle represents each genotype of the Thai-EV-Gs

We performed a survey of EV-Gs on pig farms during December 2014-January 2018. We collected 777 samples including intestinal samples (*n* = 114) and fecal samples (*n* = 663), from 73 pig farms in 20 provinces of 7 livestock regions (livestock regions 1,2,3,4,5,7 and 8). Pig samples were collected from pig farms located in 20 provinces of Thailand, including Ayutthaya, Burirum, Chachoengsao, Chaiyaphum, Chiang Rai, Chonburi, Kanchanaburi, Khon Kaen, Mukdahan, Nakhon Nayok, Nakhon Pathom, Nakhon Ratchasima, Nakhon Si Thammarat, Prachinburi, Prachuap Khiri Khan, Ratchaburi, Saraburi, Suphanburi, Trang and Ubon Ratchathani (Fig. 1). The samples were collected from 4 age groups of pigs, including suckling pigs (1 day–4 weeks) (*n* = 444), nursery pigs (5–8 weeks) (*n* = 169), fattening pigs (9–20 weeks) (*n* = 58) and breeders (boar, gilt and sow) (*n* = 106) (Table 1).

**Table 1:** Occurrences of EV-Gs by age group of pigs tested in this study

Group of pigs	Age	Number of pigs	RT-PCR for EV-Gs Positive (%)
Suckling (*n* = 444)	< 4 weeks	444	288 (64.9 %)^a, b^
Nursery (*n* = 169)	5–8 weeks	169	151 (89.4 %)^a, c^
Fattening (*n* = 63)	9–20 weeks	58	52 (89.7 %)^b, d^
Breeder (*n* = 106)	boar, gilt and sow	106	65 (61.3 %)^c, d^
		777	556/777 (71.6 %)

^a, b, c, d^Statistically significant difference between groups (*p* < 0.05)

### High occurrence of EV-Gs in pigs in Thailand

RT-PCR specific to the 5’UTR was used for the detection of EV-Gs. Our results showed that the occurrence of EV-Gs was 71.6 % (556/777). Based on the three-year survey, EV-GS could be detected year-round in almost every month and in all livestock regions (50–100 %), where 2 livestock regions (1 and 5) were 100 % positive for EV-Gs (Supplement Fig. 1). EV-Gs could be detected from 52 out of 77 pig farms (71.2 %). The occurrence of EV-Gs was highest in the fattening group (89.7 %; 52/58), followed by the nursery group (89.4 %; 151/169), suckling group (64.9 %; 288/444) and breeder group (61.3 %; 65/106). EV-Gs positive rates in the nursery (89.4 %) and fattening (89.7 %) groups were significantly higher than those in the suckling (64.9 %) and breeder (61.3 %) groups, but there was no significant difference between the nursery and fattening groups (Tables 1 and 2).

**Table 2:** Occurrences of EV-Gs tested in this study

Province	Livestock region	# of farm	# of pigs	RT-PCR for EV-Gs	
				positive farm (%)	positive sample (%)
Ayutthaya	1	1	15	1 (100%)	15 (100%)
Saraburi	1	1	3	1 (100%)	3 (100%)
Chachoengsao	2	4	24	4 (100%)	18/24 (75.0%)
Chonburi	2	7	54	4 (57.1%)	24/54 (44.4%)
Nakhon Nayok	2	2	12	1 (50.0%)	1/12 (8.3%)
Prachinburi	2	3	78	3 (100%)	55/78 (70.5%)
Burirum	3	1	5	1 (100%)	5 (100%)
Chaiyaphum	3	1	2	0 (0%)	0 (0%)
Nakhon Ratchasima	3	9	417	5 (55.6%)	330 (79.1%)
Ubon Ratchathani	3	2	8	1 (50.0%)	1 (12.5%)
Khon Kaen	4	1	11	1 (100%)	11 (100%)
Mukdahan	4	1	3	0 (0%)	0 (0%)
Chiang Rai	5	1	1	1 (100%)	1 (100%)
Kanchanaburi	7	2	10	2 (100%)	6 (60.0%)
Nakhon Pathom	7	10	30	7 (70.0%)	11 (36.7%)
Prachuap Khiri Khan	7	3	27	3 (100%)	26 (96.3%)
Ratchaburi	7	14	41	10 (71.4%)	20 (48.8%)
Suphanburi	7	3	7	2 (66.7%)	3 (42.9%)
Nakhon Si Thammarat	8	1	2	0 (0%)	0 (0%)
Trang	8	2	16	2 (100%)	16 (100%)
N/A^a^	N/A	4	11	3 (75.0%)	10 (90.9%)
		73	777	52 (71.2%)	556 (71.6%)

^a^*N/A* not available

In this study, the co-circulation of EV-Gs with other enteric swine viruses was analyzed. We found co-circulation of EV-G, porcine epidemic diarrhea virus (PEDV) and porcine deltacoronavirus (PDCoV) (0.1 %); PDCoVs/EV-Gs (1.7 %); and PEDVs/EV-Gs (30.4 %). In detail, for the suckling group (*n* = 444), the co-circulation of PEDVs/EV-Gs (29.7 %) and PDCoVs/EV-Gs (0.9 %) was observed. For the nursery group (*n* = 169), the co-circulation of PEDVs/EV-Gs (39.1 %) and PDCoVs/EV-Gs (1.78 %) was observed. For the fattening group (*n* = 58), we observed the co-circulation of PEDVs/EV-Gs (29.3 %) and PDCoVs/EV-Gs (6.9 %). For the breeder group (*n* = 106), we observed the co-circulation of PEDVs/EV-Gs (19.81 %) and PDCoVs/EV-Gs (1.9 %).

### Genetic characteristics of Thai EV-Gs

In this study, representative viruses for EV-Gs (*n* = 34) were selected for genetic characterization and phylogenetic analysis. The viruses were selected based on the following criteria; (1) location of the pig farms, (2) date of sample collection and (3) virus with high RNA copies (strong positive PCR amplicon) (Supplement Table 1). For genetic characterization, the VP1 nucleotide sequences of Thai-EV-Gs were aligned and compared with those of 20 genotypes of reference EV-Gs from the GenBank database (Supplement Table 2). Nucleotide sequence comparisons of 34 Thai-EV-Gs and 20 reference genotypes showed that the viruses had 55.05-79.95 % nucleotide identities. Thai EV-Gs showed the highest nucleotide similarities to EV-G of genotypes G1 (*n* = 7), G3 (*n* = 22), G4 (*n* = 1), G8 (*n* = 1), G9 (*n* = 1) and G10 (*n* = 2) (Fig. 1 and Supplement Table 3).

**Fig. 2:**
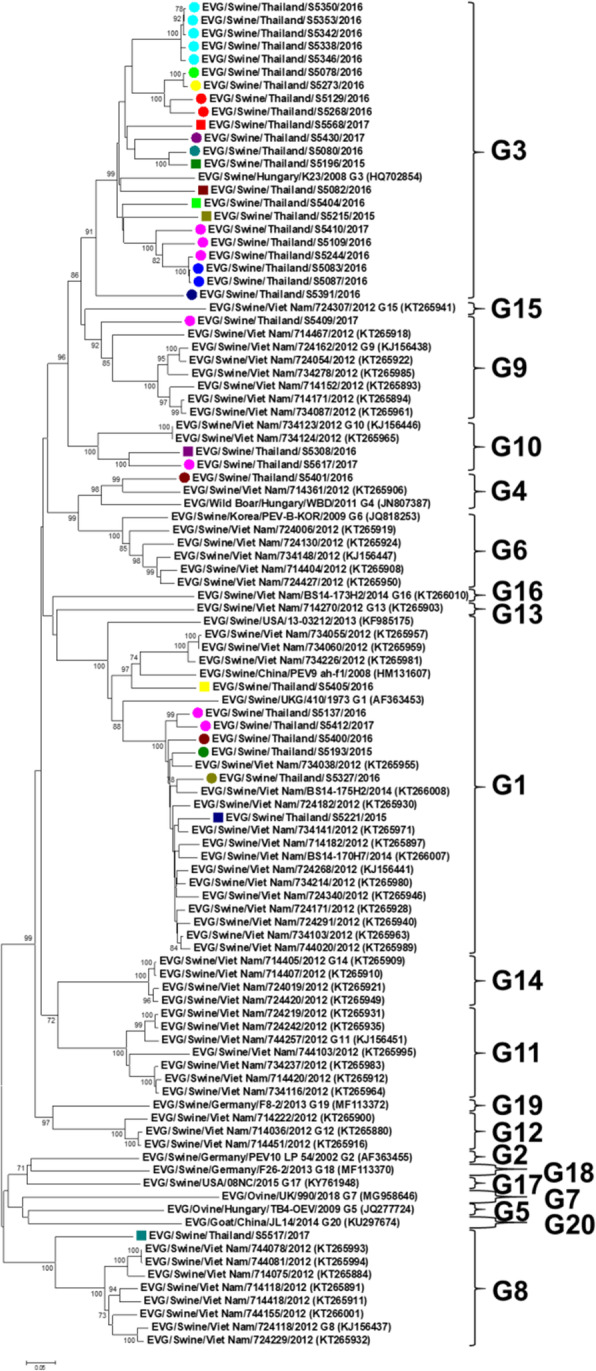
Phylogenetic analysis of the VP1 gene of the Thai-EV-Gs and reference EV-Gs genotypes. The Thai-EV-Gs are highlighted by circles and squares. Each color indicates the Thai-EV-G isolated from the same farm. The scale bar represents the distance unit between sequence pairs

The phylogenetic tree of the VP1 gene was constructed by using MEGA software version 7.0.26. The Thai-EV-Gs were collected from pigs in 2015 (*n* = 4), 2016 (*n* = 23) and 2017 (*n* = 7). The phylogenetic analysis showed that the Thai-EV-Gs were grouped with EV-Gs genotypes G1, G3, G4, G8, G9 and G10 (Fig. 2). It is noted that pig farms in 3 provinces, Nakhon Ratchasima (region 3), Ratchaburi (region 7) and Chachoengsao (region 2), harbored more than 1 genotype of EV-Gs (Fig. 1).

## Discussion

Recently, more than 20 genotypes of EV-Gs have been reported in China, Germany, Hungary, Japan, Korea, Scotland, the UK, the USA and Vietnam [5–8, 19, 20]. In infected pigs, various clinical presentations including dermatitis, poliomyelitis, pneumonia and enteritis have been observed [14, 15]. Recombination among the genotypes of EVGs has been reported; however, the presence of recombinants between human and animal enteroviruses are unclear [7, 21–23].

Most EV-Gs cause subclinical infections in pigs; for example, EV-G3, EV-G4, EV-G8, EV-G9 and EV-G10 were reported in healthy pigs in Germany, Hungary, Japan and Vietnam [4, 5, 7, 17, 18, 24–26]. On the other hand, EV-G1 (PEV-9 or PEV-B) causes diarrhea and flaccid paralysis of the hind limbs. The recombination of EV-Gs at the papain-like cysteine protease (PLCP) of Torovirus and EV-Gs genotypes G1, G2, G8, G10 and G17 has been observed, and at least two recombinant EV-Gs (type 1 and type 2) have been identified [26, 27]. Recombinant EV-G type 1 has been reported in pigs with diarrhea in Belgium, Germany, Japan and the US [17, 24, 25]. Recombinant EV-G type 2 has been identified in pigs in China [28]. Unfortunately, the genetic analysis in this study did not cover the recombination region. Therefore, a survey of intraspecies and interspecies recombination among EV-Gs should be carried out in the future.

Since epidemiological data and genetic information on EV-Gs are limited, this study provided information about the genetic diversity of EV-Gs from pig farms in Thailand. We found a high occurrence of EV-Gs, with positivity detected in 71.6 % of samples or 71.2 % of pig farms. Our findings agreed with previous reports of high occurrence of EV-Gs infection in Vietnam (81.6 %) [18]. In contrast, studies in China, the Czech Republic, Italy and Spain reported low to moderate EV-G prevalence, ranging from 0 to 50.2 % [16, 29–31]. Our results showed that Thai-EV-Gs could be detected in pigs in all livestock regions surveyed, suggesting that EV-Gs circulate throughout the country. EV-Gs positivity was high in fattening and nursery pigs, significantly higher than those in breeder and suckling pigs. Our results are consistent with a previous study showing that EV-Gs were more frequently detected in weaning pigs than in older pigs [7]. During the three-year survey, we observed that EV-Gs infection could be detected year-round (except May 2015), suggesting persistent infection. EV-Gs positivity ranged from 0 to 100 % and was high during the rainy season (84.3 %) (Supplement Table 4). The EV-G positivity during the rainy season was significantly higher than that in the summer and winter seasons, suggesting a seasonal pattern. It should be noted that a seasonal pattern of EV-Gs in pigs has never been reported before. Similarly, enterovirus infection in humans increases during the rainy season [32, 33]. In this study, the co-circulation of EV-Gs with other enteric viruses (PEDVs, PDCoVs) was observed. The co-circulation of PEDVs/EV-Gs and PDCoVs/EV-Gs was observed in all age groups.

Representative Thai-EV-Gs (*n* = 34) were selected for VP1 gene sequencing. The phylogenetic tree and nucleotide identities of the VP1 gene revealed that Thai-EV-Gs could be classified into at least 6 genotypes (EV-G1, EV-G3, EV-G4, EV-G8, EV-G9 and EV-G10). In this study, genotype classification was based on > 25 % nucleotide divergence of the VP1 nucleotide sequences [34]. EV-G3 was the predominant genotype in Thailand, which is similar to a previous survey in Japan [4]. On the other hand, EV-G1 and EV-G6 were the predominant genotypes in Vietnam [18]. Our results showed that EV-G1 was primarily detected in suckling, nursery and breeder pigs, EV-G3 could be detected in suckling pigs and breeders. EV-G9 could be detected only in suckling pigs, EV-G10 and EV-G4 could be detected only in nursery pigs, and EV-G8 could be detected only in fattening pigs. Thus, the genotypes of EV-Gs might be associated with the age of the pigs, which is in agreement with a previous study conducted in Vietnam [7].

## Conclusions

To the best of our knowledge, this is the first molecular detection and characterization of EV-Gs in pigs from Thailand. This study reported a high occurrence of EV-Gs in pigs in Thailand. EV-Gs could be detected on pig farms throughout the country. The rainy season and weaning pigs (nursery and fattening pigs) were potential risk factors for EV-G infection. At least 6 genotypes of Thai-EV-Gs circulate in Thailand. The predominant genotype was genotype G3. The prevention and control of EV-G infection should focus on potential risk factors (weaning pigs and the rainy season) along with proper farm management.

## Methods

### Sample collection from the pigs

From December 2014 to January 2018, we collected fecal samples (*n* = 663) and intestinal tissue (*n* = 114) samples from 73 pig farms in 20 provinces, including Ayutthaya, Burirum, Chachoengsao, Chaiyaphum, Chiang Rai, Chonburi, Kanchanaburi, Khon Kean, Mukdahan, Nakhon Nayok, Nakorn Pathom, Nakhon Ratchasima, Nakhon Si Thammarat, Prachinburi, Prachuap Khiri khan, Ratchaburi, Saraburi, Suphanburi, Trang and Ubon Ratchathani. The pig farms are located in 7 livestock areas of Thailand (Table 2; Fig. 1). We selected pig farms for sample collection based on the following criteria; (1) pig farms located in high-density swine production areas, (2) pig farms with a history of diarrhea outbreaks, and (3) collaboration of the farm owners. In detail, the samples (*n* = 777) were collected from pigs of different age groups, including suckling pigs (*n* = 444), nursery pigs (*n* = 169), fattening pigs (*n* = 58) and breeders (*n* = 106) (Table 1). The samples were transported on ice to the laboratory within 24 h. All of the samples were stored at -80 °C immediately until sample preparation. Written informed consent was acquired from the animal owners in this study. This study was carried out in compliance with the ARRIVE guidelines. All experimental protocols were approved by Chulalongkorn University, the Faculty of Veterinary Sciences, Animal Care and Use Committee (#IACUC 1831033).

### Identification of Enterovirus G (EV-G)

For the preparation of fecal samples, 1 g of fecal sample was diluted with 9 ml of 1X PBS to obtain a 10 % fecal suspension. For the preparation of intestinal samples, 1 g of intestinal tissue sample was homogenized with 9 ml of MEM to obtain a 10 % tissue homogenate suspension. Then, the 10 % suspension sample was centrifuged at 2,500 rpm at 4 °C for 10 min. Later, 200 µl of the supernatant was subjected to RNA extraction. Viral RNA extraction from the sample supernatant was conducted by using the Genti^TM32^ Automated nucleic acid extraction system (GeneAll® Biotechnology, Seoul, Korea) following the manufacturer’s recommendations. After automated nucleic acid extraction, the RNA samples were subjected to EV-G detection by using RT-PCR specific to the 5’UTR with a SuperScript III Platinum Taq One-Step RT-PCR kit (Invitrogen, CA, USA) [14, 35]. In brief, 30 µl of One-Step RT-PCR contained 3 µl of RNA, 1.2 µl of each forward and reverse primer, 15 µl of 2X Reaction Mix (0.4 mM of each dNTP, 6 mM MgSo4), 0.6 µl of SuperScript III RT/Platinum Taq Mix and nuclease-free water up to the reaction volume. RT-PCR conditions included reverse transcription at 55 °C for 30 min; initial denaturation at 94 °C for 2 min; 40 cycles of denaturation at 94 °C for 30 s, annealing at 55 °C for 45 s, and elongation at 72 °C for 1 min; and final elongation at 72 °C for 7 min. The PCR products were then visualized by gel electrophoresis on a 1.5 % of agarose gel in 0.5× Tris borate EDTA (TBE). The expected amplification product size was 150 base pairs.

### Genetic characterization and phylogenetic analysis of EV-Gs

To characterize the Thai-EV-Gs, viruses (*n* = 34) were selected and subjected to VP1 gene sequencing based on the representation of the locations of the pig farms, date of isolation and age group of the pigs. The VP1 gene was amplified by using PCR and oligonucleotide primer sets previously described with modifications [7]. In the first round, cDNA synthesis was performed by using the ImProm-II Reverse Transcription System (Promega, WI, USA). In brief, 20 µl of cDNA synthesis reaction contained 3 µl of RNA with 2 µl of random hexamer (incubation at 70 °C for 15 min) and 12 µl of transcription reaction mix (4 µl of 5X buffer ImProm-II Reaction buffer, 1 µl of dNTP mix, 2 µl of MgCl2, 1 µl of ImProm-II Reverse Transcriptase, 0.2 µl of RNase Inhibitor and 3.8 µl of Nuclease-Free water). The cDNA synthesis reaction involved 25 °C for 5 min, 42 °C for 60 min, 72 °C for 15 min and 25 °C for 5 min. In the second round, PCR amplification was performed in a 30 µl volume containing 2 µl of cDNA, 1.2 µl of each forward and reverse primer, 15 µl of 2X of TOPTaq Master Mix (QIAGEN), 3 µl of 10X Coral Load, and distilled water up to the reaction volume. The PCR conditions were denaturation at 94 °C for 3 min; 40 cycles of denaturation at 94 °C for 30 s, annealing at 50 °C for 45 s, and elongation at 72 °C for 2 min; and final elongation at 72 °C for 7 min. PCR amplicons were gel-purified and sequenced at 1st Base Laboratories, Kembangan, Malaysia. Nucleotide sequences were assembled and validated by using SeqMan software version 5.03 (DNASTAR Inc., Madison, WI, USA).

For pairwise comparison and genetic analysis of EV-Gs, nucleotide sequences and deduced amino acids of Thai-EV-Gs were aligned with reference EV-Gs (20 genotypes) available from the GenBank database. The reference nucleotide sequences were retrieved from the GenBank database based on the EV-G genotypes and geographical location (Supplement Table 2). The nucleotide sequences and deduced amino acids of the VP1 gene of the viruses were aligned and compared using MegAlign software v.5.03 (DNASTAR Inc., Wisconsin, USA) [36]. For phylogenetic analysis, the VP1 gene of the Thai-EV-Gs was aligned with those of reference EV-Gs and the phylogenetic tree was constructed by using MEGA version 7.0.26 with the neighbor-joining algorithm and bootstrap analysis of 1,000 replications [37].

### Statistical analysis

Statistical analysis among the age groups of pigs and EV-G positivity were analyzed by using the chi-square test. A *p*-value of < 0.05 was considered statistically significant.

## Supplementary Information

**Additional file 1: Supplement Table 1.** Detailed description of the Thai-EV-Gs characterized in this study. **Supplement Table 2.** Reference EV-Gs representing 20 genotypes included in the phylogenetic analysis. **Supplement Table 3.** Pairwise comparison of nucleotide sequences of VP1 of Thai EV-Gs with reference genotypes of EV-Gs. **Supplement Table 4.** Occurrences of EV-Gs by season in this study. **Supplement Table 5.** List of primer used in this study. **Supplement Figure 1.** Distribution of EV-Gs by provinces. The highlighted provinces represent the occurrence of EV-Gs by farms, and the number represents the occurrence of EV-Gs by samples in each province (the map of Thailand with the permission by World Trade Press).

## Data Availability

All data generated and analyzed in this study are included in this article and supplemental materials. The nucleotide sequence data that support the findings of this study are openly available in the GenBank database at https://www.ncbi.nlm.nih.gov/genbank/, accession numbers MW732933-MW732966.

## Abbreviations

EV-G: Enterovirus G
PDCoV: Porcine Deltacoronavirus
PEDV: Porcine Epidemic Diarrhea virus
PEV: Porcine enterovirus
PTV: Porcine teschovirus
PSV: Porcine sapelovirus
EV-G-PL^pro^: EV-G containing papain-like cysteine protease
