# Supplementary material for: Distribution and genetic diversity of Enterovirus G (EV-G) on pig farms in Thailand
Source: BMC Vet Res. 2021 Aug 16;17:277. doi: 10.1186/s12917-021-02988-6 (PMC8369780; doi:10.1186/s12917-021-02988-6)

**Supplement Materials**

**Distribution and genetic diversity of Enterovirus G (EV-G)**

**on pig farms in Thailand**

Taveesak Janetanakit^1,2^, Supassama Chaiyawong^1,2^, Kamonpan Charoenkul^1,2^, Ratanaporn Tangwangvivat^1^ , Ekkapat Chamsai^1^, Kitikhun Udom^1^, Waleemas Jairak^1^ and Alongkorn Amonsin^1,2*^

^1^ Center of Excellence for Emerging and Re-emerging Infectious Diseases in Animals, Faculty of Veterinary Science, Chulalongkorn University, Bangkok, Thailand

^2^ Department of Veterinary Public Health, Faculty of Veterinary Science, Chulalongkorn University, Bangkok, Thailand

*Corresponding author: Professor Dr. Alongkorn Amonsin

Mailing address: Department of Veterinary Public Health, Faculty of Veterinary Science, Chulalongkorn University, Bangkok, Thailand 10330

Phone: +66 2218 9578 Fax: +66 2218 9577

E-mail: Alongkorn.a@chula.ac.th

**Keywords:** Distribution; Diversity; Enterovirus G; Pigs; Thailand

Running Head:

Genetic diversity of EV-Gs in pigs, Thailand

# List of Authors

Taveesak Janetanakit taveesak.jane@gmail.com

Supassama Chaiyawong supasama@hotmail.com

Kamonpan Charoenkul kamonphanja15@gmail.com

Ratanaporn Tangwangvivat ratanaporn.tw@gmail.com

Ekkapat Chamsai j_ekkapat@hotmail.com

Kitikhun Udom kytykhyun@gmail.com

Waleemas Jairak waleemas.wj@gmail.com

Alongkorn Amonsin [alongkorn.a@chula.ac.th](mailto:alongkorn.a@chula.ac.th)

Supplement Table 1. Detailed description of the Thai-EV-Gs characterized in this study

| Virus ID | Farm # | Collection date | Province | Age group* | Sample type | Gene | Genotype | GenBank # |
| --- | --- | --- | --- | --- | --- | --- | --- | --- |
| S5400 | 1 | Dec-16 | Ratchaburi | Nursery | Feces | VP1 | G1 | MW732956 |
| S5401 |  | Dec-16 | Ratchaburi | Nursery | Feces | VP1 | G4 | MW732957 |
| S5193 | 18 | Feb-15 | Nakhon Pathom | Suckling | Feces | VP1 | G1 | MW732941 |
| S5327 | 20 | Nov-16 | Nakhon Pathom | Nursery | Feces | VP1 | G1 | MW732949 |
| S5391 | 25 | Nov-16 | Chonburi | Suckling | Feces | VP1 | G3 | MW732955 |
| S5430 | 32 | Feb-17 | Kanchanaburi | Suckling | Feces | VP1 | G3 | MW732963 |
| S5080 | 33 | Apr-16 | Kanchanaburi | Suckling | Small intestine | VP1 | G3 | MW732934 |
| S5129 | 34 | Jun-16 | Prachinburi | Suckling | Feces | VP1 | G3 | MW732939 |
| S5268 |  | Aug-16 | Prachinburi | Suckling | Feces | VP1 | G3 | MW732946 |
| S5078 | 35 | Apr-16 | Prachinburi | Suckling | Feces | VP1 | G3 | MW732933 |
| S5273 | 36 | Sep-16 | Prachinburi | Suckling | Feces | VP1 | G3 | MW732947 |
| S5083 | 40 | May-16 | Nakhon Ratchasima | Breeder | Feces | VP1 | G3 | MW732936 |
| S5087 |  | May-16 | Nakhon Ratchasima | Suckling | Feces | VP1 | G3 | MW732937 |
| S5109 | 41 | May-16 | Nakhon Ratchasima | Suckling | Feces | VP1 | G3 | MW732938 |
| S5137 |  | Jun-16 | Nakhon Ratchasima | Breeder | Feces | VP1 | G1 | MW732940 |
| S5244 |  | Aug-16 | Nakhon Ratchasima | Suckling | Feces | VP1 | G3 | MW732945 |
| S5409 |  | Jan-17 | Nakhon Ratchasima | Suckling | Feces | VP1 | G9 | MW732960 |
| S5410 |  | Jan-17 | Nakhon Ratchasima | Suckling | Feces | VP1 | G3 | MW732961 |
| S5412 |  | Jan-17 | Nakhon Ratchasima | Suckling | Feces | VP1 | G1 | MW732962 |
| S5617 |  | Aug-17 | Nakhon Ratchasima | Nursery | Feces | VP1 | G10 | MW732966 |
| S5338 | 43 | Nov-16 | Nakhon Ratchasima | Suckling | Feces | VP1 | G3 | MW732950 |
| S5342 |  | Nov-16 | Nakhon Ratchasima | Suckling | Feces | VP1 | G3 | MW732951 |
| S5346 |  | Nov-16 | Nakhon Ratchasima | Suckling | Feces | VP1 | G3 | MW732952 |
| S5350 |  | Nov-16 | Nakhon Ratchasima | Suckling | Feces | VP1 | G3 | MW732953 |
| S5353 |  | Nov-16 | Nakhon Ratchasima | Suckling | Feces | VP1 | G3 | MW732954 |
| S5082 | 46 | Apr-16 | Khon Kaen | Suckling | Small intestine | VP1 | G3 | MW732935 |
| S5196 | 51 | Feb-15 | Suphan Buri | Suckling | Feces | VP1 | G3 | MW732942 |
| S5215 | 53 | Dec-15 | Suphan Buri | Suckling | Feces | VP1 | G3 | MW732943 |
| S5221 | 58 | Nov-15 | Trang | Nursery | Feces | VP1 | G1 | MW732944 |
| S5308 | 63 | Oct-16 | Prachuap Khiri Khan | Nursery | Feces | VP1 | G10 | MW732948 |
| S5517 | 65 | May-17 | Chachoengsao | Fattening | Feces | VP1 | G8 | MW732964 |
| S5568 | 66 | Jun-17 | Chachoengsao | Suckling | Feces | VP1 | G3 | MW732965 |
| S5404 | 68 | Dec-16 | Nakhon Nayok | Suckling | Feces | VP1 | G3 | MW732958 |
| S5405 | 70 | Dec-16 | Chiang Rai | Nursery | Feces | VP1 | G1 | MW732959 |

*Age groups; Suckling (<4 weeks), Nursery (5-8 weeks), Fattening (9-20 weeks), Breeder (boar, gilt and sow)

Supplement Table 2. Reference EV-Gs representing 20 genotypes included in the phylogenetic analysis

| Virus strain | Genotype | GenBank # | Host |
| --- | --- | --- | --- |
| EV-G/Swine/UKG/410/1973 | G1 | AF363453 | Swine |
| EV-G/Swine/Germany/PEV10_LP_54/2002 | G2 | AF363455 | Swine |
| EV-G/Swine/Hungary/K23/2008 | G3 | HQ702854 | Swine |
| EV-G/Wild Boar/Hungary/WBD/2011 | G4 | JN807387 | Wild boar |
| EV-G/Ovine/Hungary/TB4-OEV/2009 | G5 | JQ277724 | Sheep |
| EV-G/Swine/Korea/PEV-B-KOR/2009 | G6 | JQ818253 | Swine |
| EV-G/Ovine/UK/990 | G7 | MG958646 | Sheep |
| EV-G/Swine/Vietnam/724118/2012 | G8 | KJ156437 | Swine |
| EV-G/Swine/Vietnam/724162/2012 | G9 | KJ156438 | Swine |
| EV-G/Swine/Vietnam/734123/2012 | G10 | KJ156446 | Swine |
| EV-G/Swine/Vietnam/744257/2012 | G11 | KJ156451 | Swine |
| EV-G/Swine/Vietnam/714036/2012 | G12 | KT265880 | Swine |
| EV-G/Swine/Vietnam/714270/2012 | G13 | KT265903 | Swine |
| EV-G/Swine/Vietnam/714405/2012 | G14 | KT265909 | Swine |
| EV-G/Swine/Vietnam/724307/2012 | G15 | KT265941 | Swine |
| EV-G/Swine/Vietnam/BS14-173H2/2014 | G16 | KT266010 | Swine |
| EV-G/Swine/USA/08NC/2015 | G17 | KY761948 | Swine |
| EV-G/Swine/Germany/F26-2/2013 | G18 | MF113370 | Swine |
| EV-G/Swine/Germany/F8-2/2013 | G19 | MF113372 | Swine |
| EV-G/Goat/China/JL14/2014 | G20 | KU297674 | Goat |

| Supplement Table 3. Pairwise comparison of nucleotide sequences of VP1 of Thai EV-Gs with reference genotypes of EV-Gs | | | | | | | | | | | | | | | | | | | | | |
| --- | --- | --- | --- | --- | --- | --- | --- | --- | --- | --- | --- | --- | --- | --- | --- | --- | --- | --- | --- | --- | --- |
|  | % Nucleotide identities of VP1 | | | | | | | | | | | | | | | | | | | |  |
| Genotype | G1 | G2 | G3 | G4 | G5 | G6 | G7 | G8 | G9 | G10 | G11 | G12 | G13 | G14 | G15 | G16 | G17 | G18 | G19 | G20 |  |
| G1 | 77.59% | 62.52% | 63.30% | 65.27% | 60.42% | 67.37% | 58.32% | 61.07% | 64.74% | 67.10% | 66.32% | 67.76% | 67.50% | 67.37% | 62.25% | 66.06% | 61.86% | 60.81% | 66.71% | 57.80% |  |
| G4 | 64.88% | 65.53% | 67.37% | 78.90% | 62.39% | 73.53% | 58.85% | 64.22% | 66.45% | 65.27% | 70.25% | 66.84% | 66.97% | 68.68% | 67.76% | 65.53% | 64.61% | 64.88% | 67.63% | 61.34% |  |
| G1 | 77.20% | 62.25% | 63.30% | 65.27% | 60.03% | 67.23% | 58.32% | 60.55% | 65.53% | 66.71% | 65.40% | 67.10% | 66.58% | 66.71% | 62.65% | 66.06% | 61.07% | 61.34% | 67.23% | 57.67% |  |
| G1 | 77.33% | 61.86% | 63.04% | 64.74% | 61.21% | 67.10% | 57.93% | 61.34% | 63.96% | 65.92% | 67.10% | 66.58% | 65.92% | 67.37% | 61.86% | 66.06% | 61.07% | 60.42% | 65.66% | 58.32% |  |
| G3 | 64.48% | 62.78% | 74.97% | 66.58% | 61.47% | 69.33% | 57.54% | 63.56% | 72.48% | 70.77% | 69.20% | 70.38% | 67.23% | 65.53% | 71.04% | 64.74% | 62.65% | 63.17% | 68.15% | 62.39% |  |
| G3 | 62.12% | 60.55% | 79.95% | 65.79% | 60.16% | 68.15% | 55.96% | 60.16% | 72.35% | 69.72% | 67.89% | 67.76% | 65.66% | 65.79% | 70.38% | 66.06% | 60.94% | 61.21% | 65.53% | 62.12% |  |
| G3 | 61.60% | 61.21% | 79.29% | 66.97% | 60.42% | 67.89% | 55.83% | 60.29% | 71.69% | 70.12% | 67.50% | 66.06% | 65.01% | 66.06% | 69.59% | 63.30% | 59.76% | 61.47% | 65.14% | 61.34% |  |
| G3 | 62.25% | 62.39% | 79.29% | 65.79% | 60.16% | 65.92% | 55.83% | 60.55% | 72.61% | 70.77% | 68.55% | 67.89% | 66.71% | 64.48% | 70.25% | 64.88% | 61.60% | 60.81% | 66.06% | 60.29% |  |
| G3 | 60.81% | 61.86% | 77.85% | 65.92% | 59.76% | 66.06% | 56.62% | 60.55% | 73.00% | 71.17% | 68.28% | 68.15% | 65.92% | 65.27% | 70.38% | 65.66% | 61.47% | 61.86% | 65.01% | 60.42% |  |
| G3 | 62.12% | 61.07% | 79.82% | 65.53% | 59.63% | 67.23% | 56.62% | 60.81% | 72.61% | 71.17% | 69.07% | 67.50% | 65.92% | 65.27% | 69.86% | 65.01% | 62.39% | 60.55% | 65.27% | 60.29% |  |
| G3 | 62.25% | 61.07% | 80.08% | 65.27% | 59.50% | 67.23% | 56.62% | 60.81% | 72.61% | 71.17% | 69.20% | 67.23% | 65.79% | 64.48% | 70.64% | 64.88% | 62.39% | 60.55% | 64.61% | 60.68% |  |
| G3 | 61.60% | 64.74% | 77.20% | 68.68% | 59.11% | 68.94% | 56.62% | 63.83% | 71.95% | 72.08% | 69.86% | 67.10% | 67.37% | 66.71% | 69.86% | 64.61% | 62.25% | 60.81% | 65.66% | 60.16% |  |
| G3 | 62.25% | 61.07% | 80.08% | 65.27% | 59.50% | 67.23% | 56.62% | 60.81% | 72.61% | 71.17% | 69.20% | 67.23% | 65.79% | 64.48% | 70.64% | 64.88% | 62.39% | 60.55% | 64.61% | 60.68% |  |
| G3 | 62.25% | 64.74% | 78.77% | 68.41% | 58.98% | 68.02% | 57.54% | 63.96% | 71.95% | 70.90% | 68.68% | 68.02% | 65.53% | 67.63% | 68.28% | 65.14% | 62.52% | 61.21% | 63.83% | 60.16% |  |
| G1 | 78.51% | 62.78% | 62.52% | 64.35% | 61.21% | 66.71% | 58.98% | 62.65% | 64.09% | 66.71% | 65.53% | 66.19% | 66.06% | 66.45% | 62.65% | 66.45% | 61.47% | 61.47% | 65.66% | 59.37% |  |
| G3 | 61.86% | 64.61% | 77.72% | 68.81% | 59.76% | 68.68% | 56.75% | 63.96% | 72.08% | 71.69% | 69.59% | 67.23% | 67.10% | 66.32% | 70.12% | 64.22% | 62.25% | 60.68% | 65.66% | 59.63% |  |
| G9 | 64.22% | 61.34% | 72.35% | 66.97% | 60.55% | 67.76% | 56.09% | 61.86% | 76.28% | 70.25% | 68.81% | 69.07% | 66.06% | 68.81% | 70.77% | 63.70% | 62.52% | 63.30% | 66.06% | 59.90% |  |
| G3 | 59.90% | 63.70% | 76.93% | 68.55% | 60.68% | 68.68% | 56.75% | 62.39% | 72.48% | 71.56% | 69.72% | 67.63% | 65.14% | 66.71% | 69.86% | 64.22% | 62.78% | 61.73% | 65.79% | 60.03% |  |
| G1 | 77.33% | 62.65% | 63.17% | 63.43% | 60.55% | 66.58% | 58.85% | 61.73% | 64.22% | 66.19% | 65.79% | 66.58% | 65.40% | 66.71% | 63.56% | 65.53% | 60.42% | 60.29% | 67.10% | 58.72% |  |
| G10 | 63.70% | 64.09% | 68.68% | 69.07% | 59.24% | 67.89% | 55.31% | 60.81% | 73.39% | 79.16% | 68.94% | 67.37% | 64.22% | 67.37% | 69.20% | 66.58% | 63.96% | 62.91% | 66.19% | 60.94% |  |
| G3 | 62.52% | 63.30% | 79.55% | 66.19% | 60.16% | 65.14% | 55.70% | 61.21% | 72.61% | 68.55% | 68.41% | 67.63% | 66.19% | 66.58% | 71.82% | 64.61% | 63.43% | 60.81% | 66.97% | 59.90% |  |
| G3 | 63.17% | 62.78% | 79.42% | 66.32% | 60.16% | 65.53% | 56.62% | 61.34% | 72.48% | 68.68% | 68.15% | 67.76% | 66.32% | 66.32% | 71.30% | 64.74% | 63.17% | 60.81% | 66.71% | 59.37% |  |
| G3 | 62.65% | 63.30% | 79.55% | 66.58% | 61.07% | 65.53% | 56.23% | 61.21% | 73.00% | 69.07% | 68.15% | 67.89% | 66.84% | 66.58% | 71.56% | 64.35% | 63.30% | 61.07% | 66.58% | 59.76% |  |
| G3 | 62.52% | 62.91% | 79.42% | 66.32% | 60.03% | 65.53% | 56.62% | 60.81% | 72.87% | 68.55% | 68.81% | 67.89% | 66.19% | 66.71% | 71.69% | 64.61% | 63.43% | 60.68% | 66.84% | 59.76% |  |
| G3 | 62.91% | 63.17% | 79.82% | 66.32% | 60.42% | 65.66% | 56.36% | 61.21% | 72.87% | 68.55% | 68.55% | 68.15% | 66.58% | 66.58% | 71.69% | 64.74% | 63.43% | 60.94% | 66.84% | 59.63% |  |
| G3 | 61.34% | 61.73% | 78.77% | 66.19% | 59.24% | 68.02% | 56.09% | 62.25% | 71.43% | 70.38% | 70.25% | 68.28% | 66.45% | 65.79% | 70.12% | 62.65% | 61.47% | 62.52% | 65.27% | 61.07% |  |
| G3 | 61.60% | 61.34% | 79.42% | 66.32% | 60.94% | 68.41% | 55.57% | 60.03% | 71.43% | 69.59% | 67.23% | 67.23% | 63.96% | 65.79% | 70.12% | 64.61% | 59.63% | 61.34% | 65.40% | 61.73% |  |
| G3 | 61.47% | 60.42% | 78.11% | 66.71% | 60.94% | 66.19% | 55.05% | 61.73% | 71.30% | 69.07% | 68.02% | 67.23% | 64.88% | 65.27% | 69.46% | 64.22% | 60.68% | 60.81% | 64.88% | 60.42% |  |
| G1 | 77.20% | 61.73% | 63.17% | 64.48% | 60.29% | 66.58% | 56.36% | 61.47% | 64.09% | 66.84% | 67.89% | 66.97% | 67.37% | 68.28% | 62.52% | 65.14% | 62.25% | 61.21% | 66.32% | 59.37% |  |
| G10 | 62.65% | 63.70% | 68.41% | 68.81% | 59.37% | 66.97% | 55.31% | 61.34% | 72.48% | 77.59% | 68.28% | 66.71% | 65.01% | 66.71% | 69.33% | 66.19% | 63.04% | 62.65% | 66.97% | 58.45% |  |
| G8 | 62.25% | 65.92% | 61.07% | 63.83% | 64.09% | 64.88% | 61.60% | 78.11% | 61.21% | 61.86% | 65.92% | 65.79% | 63.04% | 65.92% | 62.91% | 61.07% | 67.89% | 66.97% | 65.79% | 61.73% |  |
| G3 | 62.78% | 60.55% | 77.98% | 66.58% | 61.60% | 67.10% | 55.96% | 60.29% | 73.13% | 69.46% | 66.58% | 68.02% | 66.06% | 65.40% | 69.46% | 62.39% | 62.39% | 60.55% | 66.19% | 61.86% |  |
| G3 | 61.47% | 63.30% | 79.03% | 66.71% | 60.03% | 68.68% | 56.75% | 61.99% | 72.35% | 69.20% | 69.07% | 67.50% | 67.10% | 67.89% | 67.63% | 65.53% | 62.25% | 62.12% | 64.74% | 60.42% |  |
| G1 | 74.57% | 62.25% | 65.14% | 66.97% | 60.42% | 66.06% | 56.75% | 62.91% | 68.15% | 67.10% | 71.04% | 68.55% | 66.06% | 69.72% | 63.56% | 66.32% | 64.48% | 62.65% | 66.84% | 61.07% |  |

Supplement Table 4. Occurrences of EV-Gs by season in this study

| Season (month) | EV-G #positive/samples (%) | Percentage (%) |
| --- | --- | --- |
|  |  |  |
| Summer (16 Feb-15 May) | 85/167 | 50.9% |
| Rainy (16 May-15 Oct) | 380/451 | 84.3%* |
| Winter (16 Oct-15 Feb) | 91/159 | 57.2% |
|  | 556/777 | 71.6% |
|  |  |  |

*Statistical significance (*p<0.05*)

Supplement Table 5. List of primer used in this study

| Primer | Target gene | Nucleotide sequence | Product size | Reference |
| --- | --- | --- | --- | --- |
|  |  |  |  |  |
| EV-G-F | 5’UTR | 5’CCCTGAATGCGGCTAAT | 150 bp | Beld *et al.,* 2004 |
| EV-G-R |  | 5’ATTGTCACCATAAGCAGCC |  | Palmquist *et al.,* 2002 |
|  |  |  |  |  |
| EV-G-VP1F | VP1 | 5’GCTGGKTATRTKACYGGDTGGTWYC | 1000 bp | Modified from  Van Dung *et al,* 2014 |
| EV-G-VP1R |  | 5’TCTTCCCAYTCDASRTTBTCCCA |  |  |

Supplement Figure 1. Distribution of EV-Gs by provinces. The highlighted provinces represent the occurrence of EV-Gs by farms, and the number represents the occurrence of EV-Gs by samples in each province (the map of Thailand with the permission by World Trade Press).

.


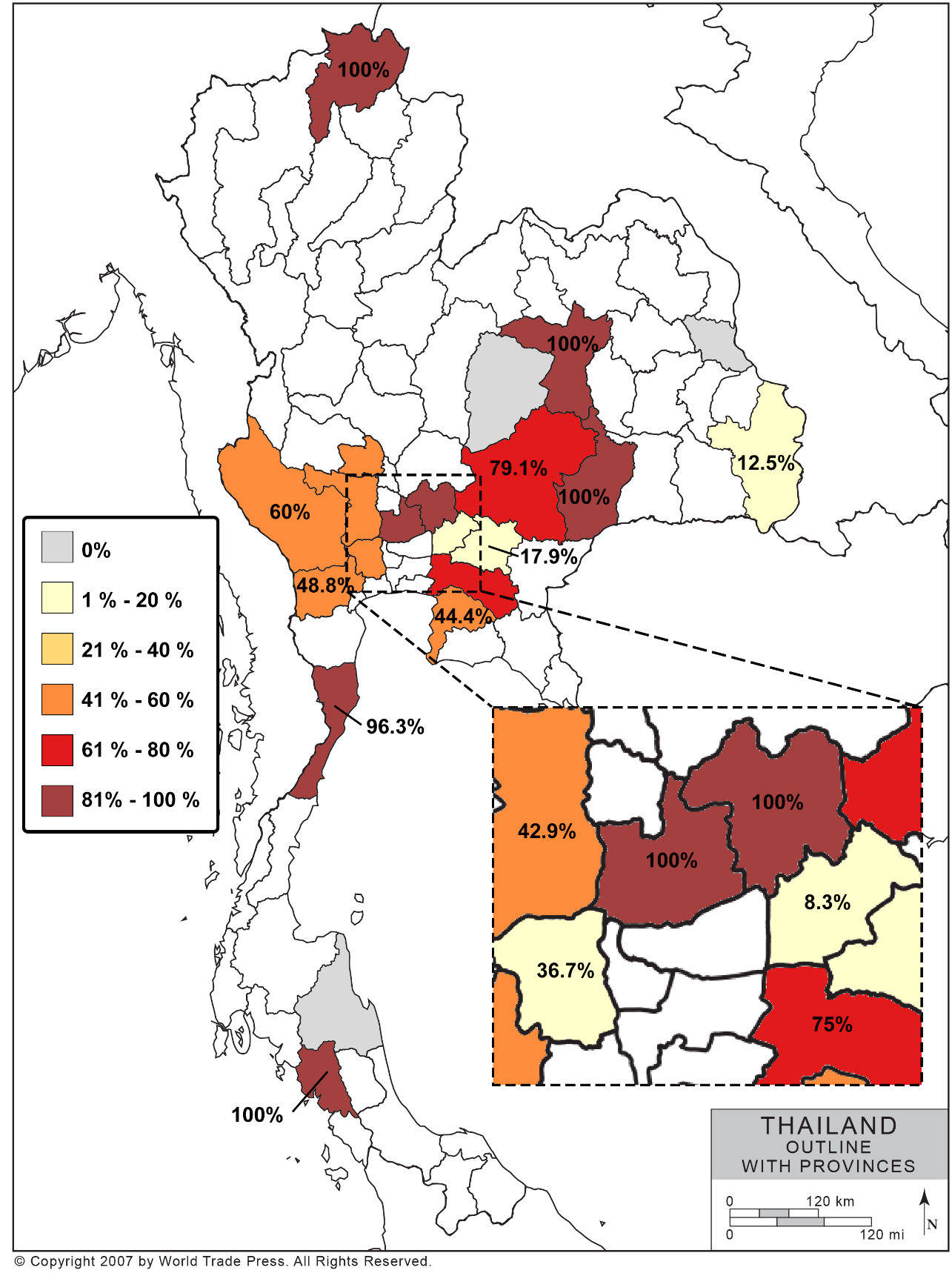

Supplement: Supplementary file 1 — Additional file 1: Supplement Table 1. Detailed description of the Thai-EV-Gs characterized in this study. Supplement Table 2. Reference EV-Gs representing 20 genotypes included in the phylogenetic analysis. Supplement Table 3. Pairwise comparison of nucleotide sequences of VP1 of Thai EV-Gs with reference genotypes of EV-Gs. Supplement Table 4. Occurrences of EV-Gs by season in this study. Supplement Table 5. List of primer used in this study. Supplement Figure 1. Distribution of EV-Gs by provinces. The highlighted provinces represent the occurrence of EV-Gs by farms, and the number represents the occurrence of EV-Gs by samples in each province (the map of Thailand with the permission by World Trade Press). [file 12917_2021_2988_MOESM1_ESM.docx]
